# Aberrant *FAM64A* mRNA expression is an independent predictor of poor survival in pancreatic cancer

**DOI:** 10.1371/journal.pone.0211291

**Published:** 2019-01-29

**Authors:** Yan Jiao, Zhuo Fu, Yanqing Li, Wei Zhang, Yahui Liu

**Affiliations:** 1 Department of Hepatobiliary and Pancreatic Surgery, The First Hospital of Jilin University, Changchun, Jilin, P.R. China; 2 Department of Hand and Foot surgery, The First Hospital of Jilin University, Changchun, Jilin, P.R. China; 3 Department of Pathophysiology, College of Basic Medical Sciences, Jilin University, Changchun, Jilin, P.R. China; University of South Alabama Mitchell Cancer Institute, UNITED STATES

## Abstract

*FAM64A*, a marker of cell proliferation, has been investigated as a potential biomarker in several cancers. In the present study, we examined the value of *FAM64A* expression in the diagnosis and prognosis of pancreatic cancer through bioinformatics analysis of data obtained from The Cancer Genome Atlas (TCGA) database. The diagnostic value of *FAM64A* expression in pancreatic cancer tissue was deteremined through receiver operating characteristic (ROC) curve analysis, and based on the obtained cut-off value, patients were divided into two groups (high *FAM64A* expression and low *FAM64A* expression). Chi-square and Fisher exact tests were applied to identify associations between *FAM64A* expression and clinical features. Moreover, the effect of *FAM64A* expression in the survival of pancreatic cancer patients was observed by Kaplan-Meier and Cox analyses. Gene set enrichment analysis (GSEA) was performed using the TCGA dataset. Our results showed that high *FAM64A* expression in pancreatic cancer was associated with survival status, overall survival (OS), and recurrence. The area under the ROC curve was 0.736, which indicated modest diagnostic value. Patients with higher *FAM64A* expression had significantly shorter OS and recurrence-free survival (RFS) times. Multivariate survival analysis demonstrated that high *FAM64A* expression was an independent risk factor for OS and RFS. GSEA identified mitotic spindles, myc targets, MTORC1 signaling, G2M checkpoint, E2F targets, DNA repair, glycolysis and unfolded protein response as differentially enriched with the high *FAM64A* expression phenotype. In conclusion, high *FAM64A* mRNA expression is an independent risk factor for poor prognosis in pancreatic cancer.

## Introduction

Pancreatic cancer is associated with a high mortality rate worldwide. Although the global incidence is relatively low (about 8/100000 persons per year) [1], the 5-year survival rate is less than 5%. The poor survival rate is, in part, due to late detection of advanced stage disease, and thus, methods for early and accurate diagnosis of pancreatic cancer would help to improve outcomes for these patients. Currently, the diagnosis of pancreatic cancer is mainly based on imaging analyses, including computed tomography (CT) and magnetic resonance imaging (MRI). Unfortunately, even these imaging modalities are ineffective in some cases of pancreatic cancer [2]. Therefore, the identification of a reliable biomarker for pancreatic cancer diagnosis and prognosis has clinical significance.

Genome changes and self-sufficiency in growth signals are among the essential alterations that lead to malignant proliferation [3]. FAM64A, also referred to as PICALM Interacting Mitotic Regulator (gene symbol, *PIMREG*) [4], was first identified as a CALM (Clathrin Assembly Lymphoid Myeloid leukemia) interacting protein, expressed in response to mitogens. Expression of FAM64A influences the subcellular localization of CALM/AF10 and antagonizes the transactivation function of the leukemic fusion protein [5–7]. As a marker of cell proliferation, FAM64A protein expression is cell cycle-dependent [6, 8], and its expression level has been studied as a potential biomarker in several cancers in recent years [6, 9–12]. However, the potential roles of *FAM64A* expression in the diagnostic and prognostic evaluation of patients with pancreatic cancer have not yet been determined.

In the present study, we compared *FAM64A* mRNA expression between pancreatic cancer patients and healthy individuals. From an analysis of the diagnostic value of *FAM64A*, we divided patients into high and low *FAM64A* expression groups and searched for correlations between *FAM64A* expression and clinical features of pancreatic cancer as well as with patients’ overall survival (OS) and recurrence-free survival (RFS). Gene set enrichment analysis (GSEA) was performed to identify the signaling pathways related to the regulatory mechanism of *FAM64A*. Our results indicate that *FAM64A* may be a useful biomarker for the diagnosis and prognosis of pancreatic cancer.

## Materials and methods

### Data mining

Level 3 expression data were downloaded from The Cancer Genome Atlas (TCGA) database and estimated as log2(x+1) transformed RSEM normalized counts. Clinical information was also obtained from TCGA database. Expression data for normal pancreatic tissue was download from The Genotype-Tissue Expression (GTEx) database. All data were processed using R software (version 3.5.1) [13].

### Statistical analysis

The expression of *FAM64A* in patients in the TCGA-Liver Hepatocellular Carcinoma (LIHC) dataset was evaluated using box plots. A received operating characteristic (ROC) curve was generated to evaluate the diagnostic value of *FAM64A* expression using the pROC package [14], and the area under curve (AUC) of ROC curve represents the diagnostic value. Patients were divided into two groups (high and low *FAM64A* expression) according to the threshold value identified from the ROC curve. The chi-square and Fisher exact tests were applied to identify correlations between *FAM64A* mRNA expression and clinical features of pancreatic cancer. OS and RFS were compared between the high and low *FAM64A* expression groups via Kaplan-Meier analysis, with p-values calculated by the log-rank test, using the Survival package in R [15, 16]. Univariate Cox analysis was performed to select potential prognostic factors, and multivariate Cox analysis was performed to verify the correlations between *FAM64A* expression and survival along with other clinical features. P<0.05 was considered statistically significant.

### GSEA

To identify potential mechanisms underlying the influence of *FAM64A* expression on pancreatic cancer prognosis, GSEA was performed to detect whether an a priori defined set of genes showed statistically significant differential expression between the high and low *FAM64A* expression groups [17, 18]. Gene sets with a normal P-value <0.05 and false discovery rate (FDR) <0.25 were considered to be significantly enriched.

## Results

### Characteristics of the study population

The clinical data of 179 patients were downloaded from TCGA database, including patients’ age, gender, and alcohol consumption history as well as the anatomic location, histological type, histologic grade, clinical stage, TNM classification, residual tumor, therapy, survival status, and recurrence of pancreatic cancer (Table 1).

**Table 1:** Clinical characteristics of the pancreatic cancer patients.

Characteristic	n (%)
Age	
<55 years	34(18.99)
≥55 years	145(81.01)
Gender	
Female	80(44.69)
Male	99(55.31)
Alcohol consumption history	
No	65(36.31)
Yes	102(56.98)
Not available	12(6.7)
Anatomic location	
Head of pancreas	139(77.65)
Body of pancreas	14(7.82)
Tail of pancreas	15(8.38)
Other	11(6.15)
Histological type	
Adenocarcinoma other subtype	26(14.53)
Adenocarcinoma ductal type	147(82.12)
Colloid carcinoma	4(2.23)
Undifferentiated carcinoma	1(0.56)
Not available	1(0.56)
Histological grade	
G1	31(17.32)
G2	96(53.63)
G3	48(26.82)
G4	2(1.12)
GX	2(1.12)
Stage	
I	21(11.73)
II	147(82.12)
III	4(2.23)
IV	5(2.79)
Not available	2(1.12)
T classification	
T1	7(3.91)
T2	24(13.41)
T3	143(79.89)
T4	3(1.68)
TX	1(0.56)
Not available	1(0.56)
N classification	
N0	50(27.93)
N1	124(69.27)
NX	4(2.23)
Not available	1(0.56)
M classification	
M0	80(44.69)
M1	5(2.79)
MX	94(52.51)
Residual tumor	
R0	107(59.78)
R1	53(29.61)
R2	5(2.79)
RX	4(2.23)
Not available	10(5.59)
Survival status	
Death	93(51.96)
Survival	86(48.04)
Recurrence	
No	98(54.75)
Yes	58(32.40)
Not available	23(12.85)

### High *FAM64A* expression in pancreatic cancer

**Fig 1:**
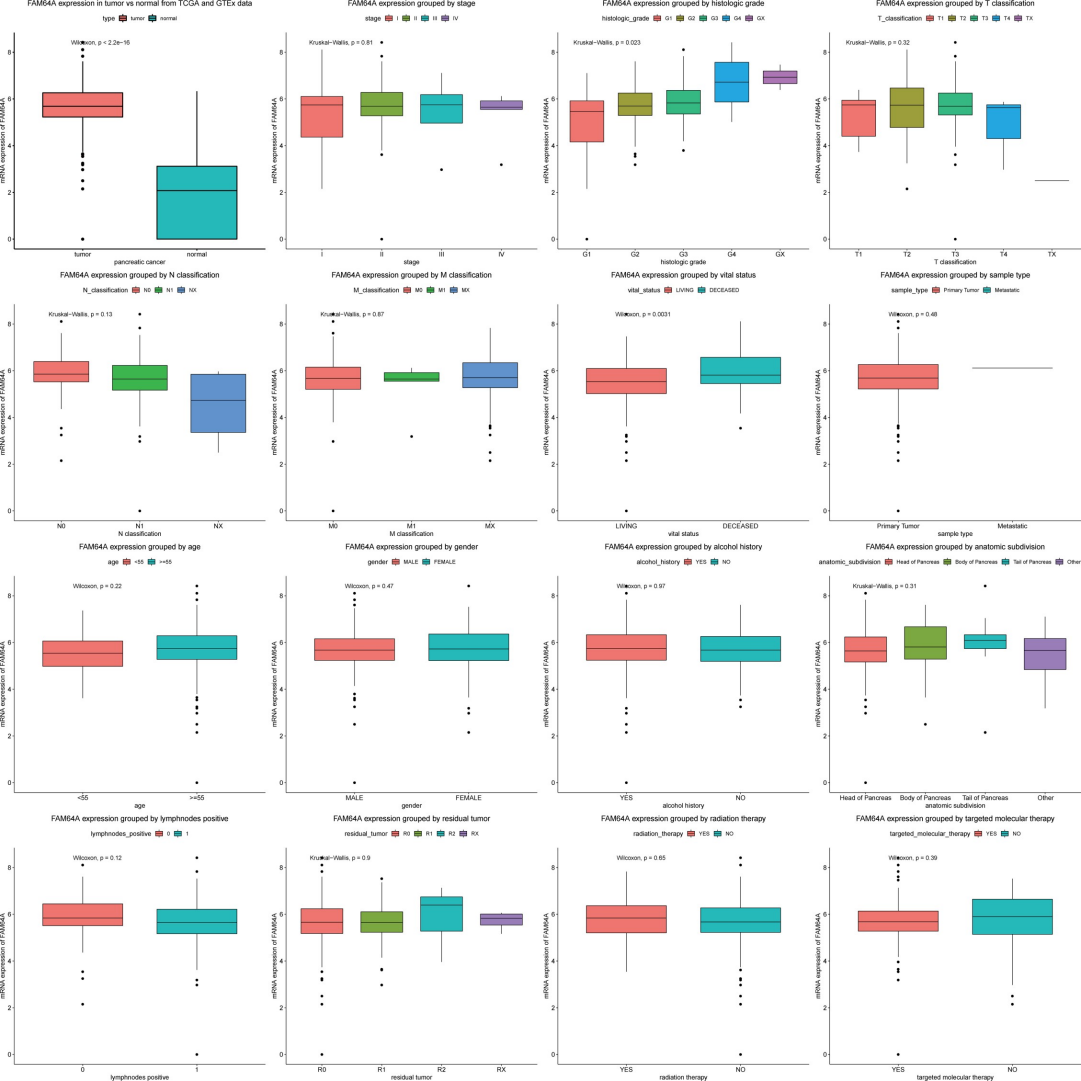
*FAM64A* expression in pancreatic cancer. *FAM64A* expression was compared between normal tissues and pancreatic cancer tissues as well as according to the cancer stage, histologic grade, anatomic location, TNM classification, survival status, sample type, patient age, gender, alcohol consumption history, lymph node positivity, residual tumor, treatment with radiation, and treatment with targeted molecular therapy.

*FAM64A* expression in pancreatic cancer and normal tissues was compared (Fig 1), and the results indicated that *FAM64A* expression was elevated in pancreatic cancer (P<2.2e-16). Moreover, differences in *FAM64A* expression were observed according to histological grade (P = 0.029) and survival status (P = 0.0021).

### Diagnostic value of *FAM64A* expression in pancreatic cancer

**Fig 2:**
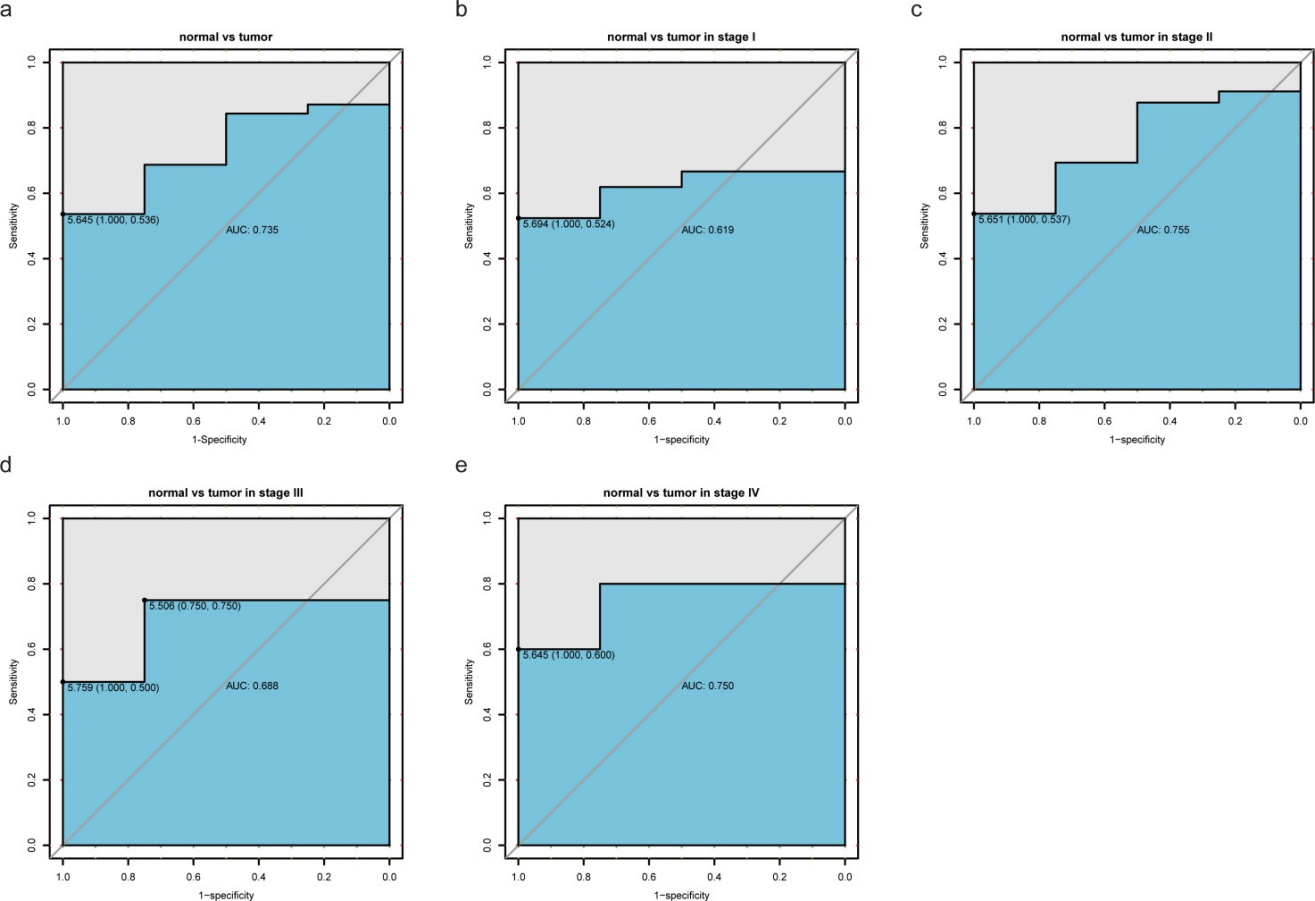
Diagnosis value of *FAM64A* expression in pancreatic cancer. (A) ROC curve for *FAM64A* expression in normal pancreatic tissue and pancreatic cancer. (B-E) Subgroup analysis for stage I, II, III, and IV pancreatic cancer.

To assess the diagnostic value of *FAM64A*, we generated a ROC curve using the expression data from pancreatic cancer patients and healthy individuals (Fig 2A). The area under the ROC curve (AUC) was 0.735, which indicated modest diagnostic value. Subgroup analysis demonstrated the diagnostic value of *FAM46A* expression in different stages of pancreatic cancer, with AUC values of 0.619 for stage I disease, 0.755 for stage II disease, 0.688 for stage III disease, and 0.750 for stage IV disease (Fig 2B–2E).

### Correlation between *FAM64A* expression and clinical features of pancreatic cancer

We divided patients into two groups (high versus low *FAM64A* expression) according to the threshold determined from the ROC curve. The associations identified between *FAM64A* expression and the clinical characteristics of pancreatic cancer cases are summarized in Table 2. Survival status (P = 0.0023) and recurrence (P = 0.0376) were significantly correlated with *FAM64A* expression.

**Table 2:** Relationship between the clinical features of pancreatic cancer and *FAM64A* expression.

Features	Variable	n	*FAM64A* (%)				χ^2^	P
			High		Low			
Age	<55 years	34	18	-15.38	16	-25.81	2.224	0.136
	≥55 years	145	99	-84.62	46	-74.19		
Gender	Female	80	55	-47.01	25	-40.32	0.487	0.485
	Male	99	62	-52.99	37	-59.68		
Alcohol use history	No	65	43	-39.81	22	-37.29	0.024	0.878
	Yes	102	65	-60.19	37	-62.71		
Anatomic location	Body of pancreas	14	9	-7.69	5	-8.06	3.288	0.349
	Head of pancreas	139	88	-75.21	51	-82.26		
	Other	11	7	-5.98	4	-6.45		
	Tail of pancreas	15	13	-11.11	2	-3.23		
Histological type	Adenocarcinoma Other subtype	26	14	-12.07	12	-19.35	2.319	0.509
	Adenocarcinoma ductal type	147	98	-84.48	49	-79.03		
	Colloid carcinoma	4	3	-2.59	1	-1.61		
	Undifferentiated carcinoma	1	1	-0.86	0	0		
Histologic grade	G1	31	16	-13.68	15	-24.19	4.334	0.363
	G2	96	65	-55.56	31	-50		
	G3	48	33	-28.21	15	-24.19		
	G4	2	1	-0.85	1	-1.61		
	GX	2	2	-1.71	0	0		
Stage	I	21	12	-10.34	9	-14.75	1.290	0.732
	II	147	97	-83.62	50	-81.97		
	III	4	3	-2.59	1	-1.64		
	IV	5	4	-3.45	1	-1.64		
T classification	T1	7	4	-3.45	3	-4.84	3.799	0.434
	T2	24	13	-11.21	11	-17.74		
	T3	143	97	-83.62	46	-74.19		
	T4	3	2	-1.72	1	-1.61		
	TX	1	0	0	1	-1.61		
N classification	N0	50	38	-32.48	12	-19.67	3.507	0.173
	N1	124	77	-65.81	47	-77.05		
	NX	4	2	-1.71	2	-3.28		
M classification	M0	80	50	-42.74	30	-48.39	0.877	0.645
	M1	5	4	-3.42	1	-1.61		
	MX	94	63	-53.85	31	-50		
Residual tumor	R0	107	68	-62.39	39	-65	0.334	0.954
	R1	53	35	-32.11	18	-30		
	R2	5	3	-2.75	2	-3.33		
	RX	4	3	-2.75	1	-1.67		
Survival status	Death	93	71	-60.68	22	-35.48	9.325	**0.002**
	Survival	86	46	-39.32	40	-64.52		
Recurrence	No	98	55	-56.12	43	-74.14	4.321	**0.038**
	Yes	58	43	-43.88	15	-25.86		

Bold values indicate P<0.05.

### High *FAM64A* expression is an independent risk factor for OS among pancreatic cancer patients

**Fig 3:**
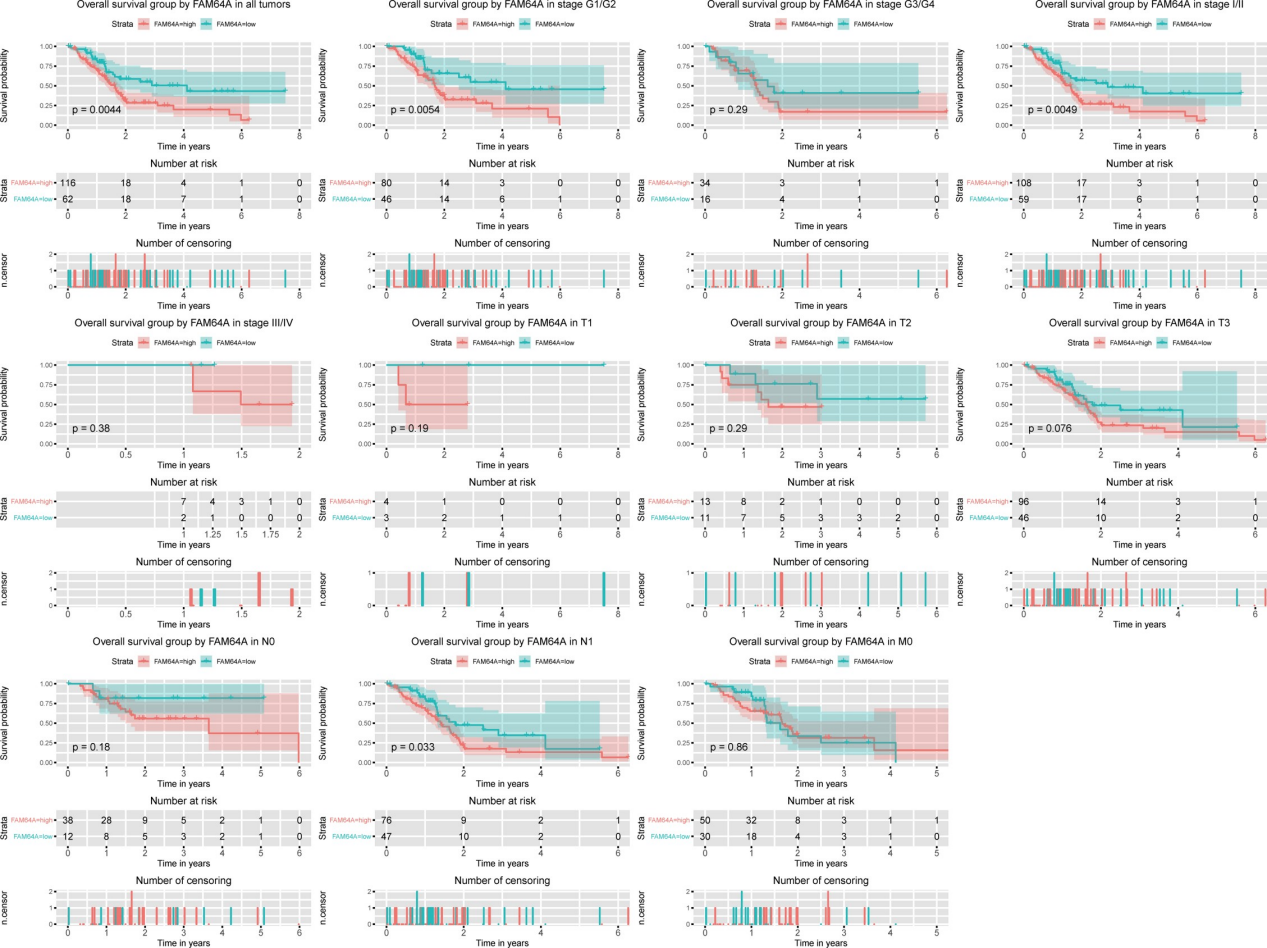
Kaplan-Meier curves for OS in pancreatic cancer. Kaplan-Meier curves for OS in pancreatic cancer for all cases and cases of histologic grade G1/G2 and G3/G4; clinical stage Ⅰ/Ⅱ, and Ⅲ/Ⅳ; and TNM classification T1, T2, T3, N0, N1, and M0.

Kaplan-Meier curves showed that high *FAM64A* expression was associated with worse OS (P = 0.0044; Fig 3). Subgroup analysis indicated that high *FAM64A* expression significantly affected the OS in pancreatic cancer cases of histological grade G1/G2 (p = 0.0054), clinical stage Ⅰ/Ⅱ (p = 0.0049), and N1 (p = 0.033). Univariate and multivariate Cox analyses showed that *FAM64A* expression was an independent risk factor for OS among pancreatic cancer patients (hazard ratio [HR] = 1.87, 95% confidence interval [CI]: 1.14–3.06, P = 0.013, Table 3).

**Table 3:** Univariate analysis and multivariate analysis of the correlation of *FAM64A* expression with OS among pancreatic cancer patients.

Parameter	Univariate analysis			Multivariate analysis		
	HR	95% CI	P	HR	95% CI	P
Age	1.22	0.73–2.05	0.446			
Gender	0.81	0.54–1.22	0.320			
Alcohol use history	1.09	0.79–1.5	0.604			
Histological type	2.07	1.36–3.16	**0.001**	1.71	1.09–2.7	**0.020**
Histologic grade	1.33	1.04–1.7	**0.021**	1.18	0.9–1.55	0.223
Stage	1.33	0.96–1.84	0.089			
T classification	1.64	1.08–2.49	**0.021**	1.11	0.68–1.8	0.684
N classification	1.93	1.23–3.03	**0.005**	1.58	0.97–2.58	0.064
M classification	1.13	0.79–1.64	0.500			
Residual tumor	1.36	1.06–1.76	**0.018**	1.43	1.1–1.86	**0.008**
*FAM64A*	1.99	1.23–3.22	**0.005**	1.87	1.14–3.06	**0.013**

Bold values indicate P<0.05. HR, hazard ratio; CI, confidence interval.

### High *FAM64A* expression is an independent risk factor for RFS in pancreatic cancer patients

**Fig 4:**
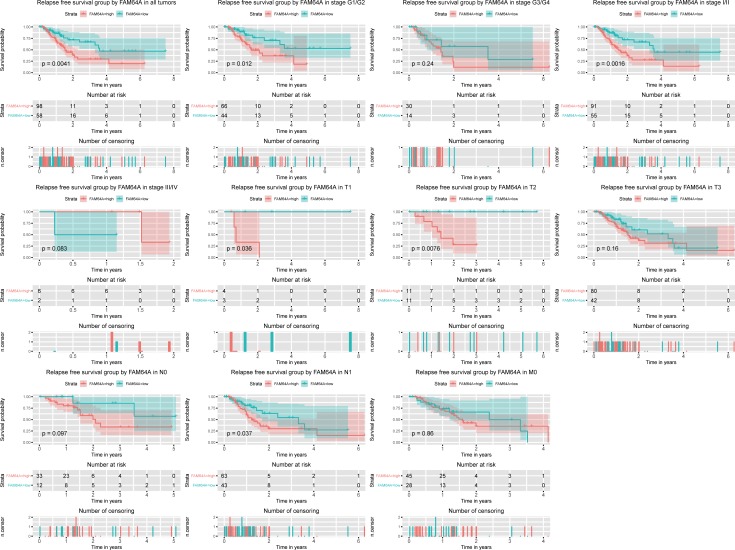
Kaplan-Meier curves for RFS in pancreatic cancer. Kaplan-Meier curves for RFS in all pancreatic cancer cases as well as cases of histologic grade G1/G2 and G3/G4; clinical stage Ⅰ/Ⅱ and Ⅲ/Ⅳ; and TNM classification T1, T2, T3, N0, N1, and M0.

Kaplan-Meier curves for RFS showed that high *FAM64A* expression was associated with poor RFS (P = 0.0041; Fig 4). Subgroup analysis identified that high *FAM64A* expression correlated significantly with poor RFS in pancreatic cancer cases of histologic grade G1/G2 (p = 0.012), clinical stage Ⅰ/Ⅱ (p = 0.0016), T1 (p = 0.036), T2 (p = 0.0076), and N1 (p = 0.037). Univariate and multivariate Cox analyses confirmed the role of *FAM64A* expression as an independent risk factor for RFS in pancreatic cancer patients (HR = 2.5, 95%CI: 1.36–4.61, P = 0.003, Table 4).

**Table 4:** Univariate analysis and multivariate analysis of the correlation of *FAM64A* expression with RFS among pancreatic cancer patients.

Parameter	Univariate analysis			Multivariate analysis		
	HR	95% CI	P	HR	95% CI	P
Age	0.93	0.51–1.71	0.827			
Gender	1.12	0.67–1.89	0.667			
Alcohol history	1.22	0.79–1.87	0.364			
Histological type	1.09	0.65–1.84	0.733			
Histologic grade	1.56	1.13–2.17	**0.007**	1.51	1.07–2.14	**0.020**
Stage	1.41	0.98–2.04	0.064			
T classification	1.51	0.94–2.41	0.088			
N classification	2.03	1.17–3.52	**0.012**	1.87	1.06–3.3	**0.032**
M classification	1.42	0.92–2.21	0.116			
Residual tumor	1.73	1.23–2.42	**0.002**	1.87	1.33–2.64	**0.000**
*FAM64A*	2.34	1.29–4.26	**0.005**	2.50	1.36–4.61	**0.003**

Bold values indicate P<0.05. HR, hazard ratio; CI, confidence interval.

### GSEA identifies an *FAM64A*-related signaling pathway

**Fig 5:**
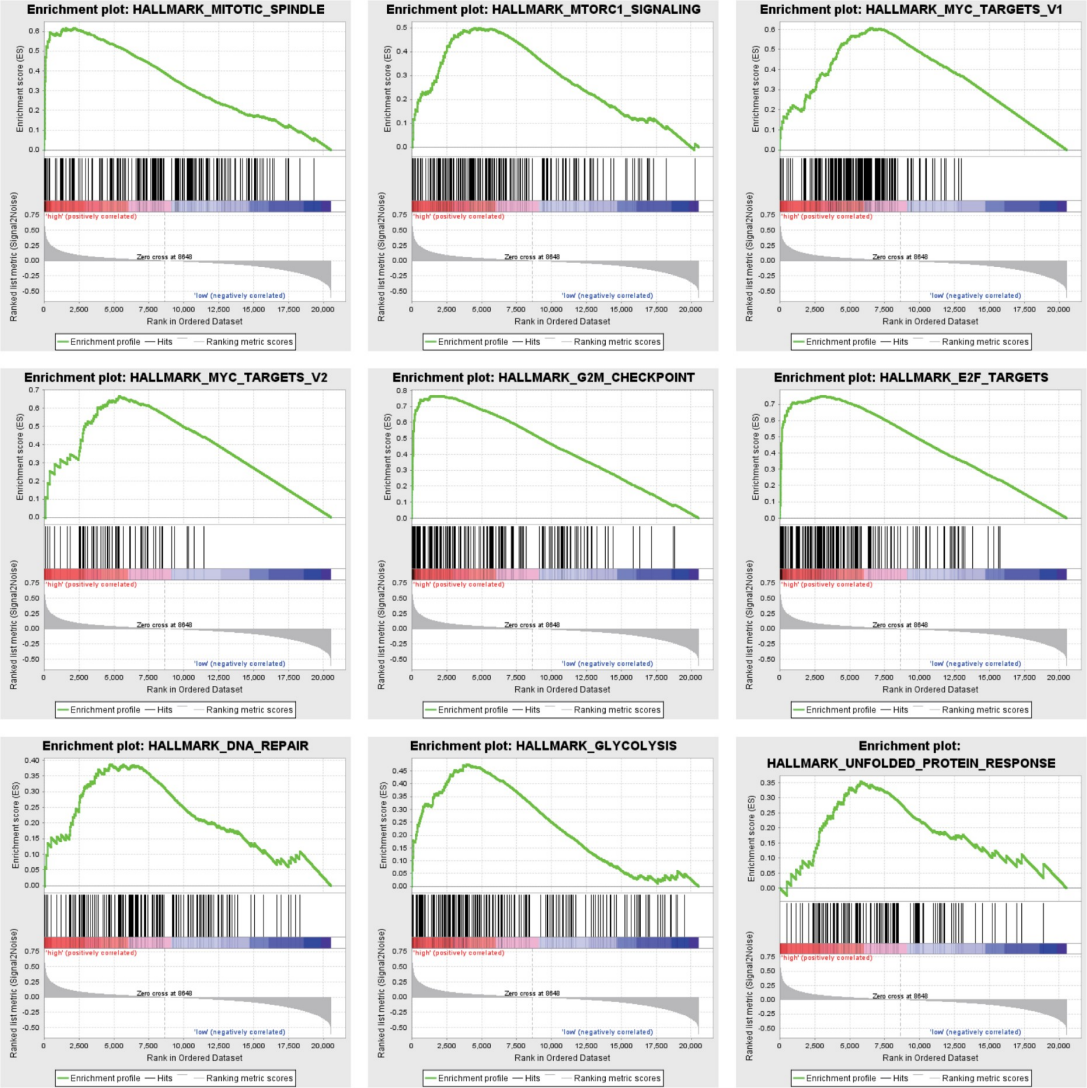
Enrichment plots from GSEA. GSEA results showing differential enrichment of genes related to mitotic spindles, myc targets, MTORC1 signaling, G2M checkpoint, E2F targets, DNA repair, glycolysis and unfolded protein response in pancreatic cancer cases with high *FAM64A* expression.

To identify signaling pathways activated in pancreatic cancer, we performed GSEA comparing the low and high *FAM64A* expression datasets. GSEA revealed significant differences (FDR <0.25, NOM P-value <0.05) in the enrichment of MSigDB Collection (h.all.v6.2.symbols.gmt), and the details are provided in Table 5. Gene sets related to mitotic spindles, myc targets, MTORC1 signaling, G2M checkpoint, E2F targets, DNA repair, glycolysis and unfolded protein response were differentially enriched with the high *FAM64A* expression phenotype (Fig 5).

**Table 5:** Gene sets enriched in the high *FAM64A* expression phenotype.

Gene set name	NES	NOM p-val	FDR q-val
HALLMARK_MITOTIC_SPINDLE	2.133	0.000	0.018
HALLMARK_MYC_TARGETS_V2	2.065	0.004	0.016
HALLMARK_MTORC1_SIGNALING	2.033	0.004	0.012
HALLMARK_MYC_TARGETS_V1	1.983	0.004	0.015
HALLMARK_G2M_CHECKPOINT	1.982	0.000	0.012
HALLMARK_E2F_TARGETS	1.930	0.000	0.015
HALLMARK_DNA_REPAIR	1.928	0.006	0.013
HALLMARK_GLYCOLYSIS	1.803	0.000	0.025
HALLMARK_UNFOLDED_PROTEIN_RESPONSE	1.772	0.049	0.026

NES: normalized enrichment score; NOM: nominal; FDR: false discovery rate. Gene sets with NOM P-value <0.05 and FDR q-value <0.25 were considered as significantly enriched.

## Discussion

Our study found that the high *FAM64A* expression was associated with poor survival status and recurrence in pancreatic cancer. Kaplan-Meier curves for OS and RFS also showed that higher expression of *FAM64A* was associated with worse outcomes in pancreatic cancer patients. Univariate and multivariate Cox analyses indicated the *FAM64A* mRNA expression may be a useful biomarker for pancreatic cancer prognosis, and ROC analysis confirmed the diagnostic value of *FAM64A* expression in pancreatic cancer.

In the field of oncology, Archangelo et al. first reported the high *FAM64A* expression in hematologic carcinomas, including leukemia and lymphoma [6]. However, few studies have investigated *FAM64A* expression in solid tumors. Zhang et al. [11] and Yamada et al. [12] found that *FAM64A* is upregulated in triple negative breast cancer and renal cell carcinoma, respectively. The present study demonstrated high *FAM64A* expression in pancreatic cancer, which is consistent with other findings for *FAM64A* expression in tumors. ROC analysis provided evidence that *FAM64A* could be developed as a biomarker for the diagnosis of pancreatic cancer, and we found that distinct histologic grades and survival status were associated with *FAM64A* expression, which suggests a possible relationship between *FAM64A* expression and survival in pancreatic cancer.

Many studies have reported that *FAM64A* plays an important role in malignant transformation. Archangelo et al. also found that the expression of *FAM64A* is elevated during mouse embryogenesis [6]. Hashimoto et al. showed that knockdown of the *FAM64A* gene in fetal cardiomyocytes leads to repression of cell cycle genes and Ki67 downregulation [19]. Barbutti et al. demonstrated that cell proliferation and cell cycle progression are correspondingly reduced upon silencing of *FAM64A* in the U937 cell line or MDA-MB-231 cells [9]. Based on these reports, higher expression of *FAM64A* indicates the promotion of cell proliferation, which might contribute to worse outcomes in pancreatic cancer patients. Yamada et al. also demonstrated the value of *FAM64A* expression in the prognosis of breast cancer and renal cell carcinoma [12]. Hu et al. explored the prognostic role of *FAM64A* expression in a variety of cancer types, including pancreatic cancer [10]. Consistently, our results showed that *FAM64A* expression was correlated with OS in patients with pancreatic cancer, and the potential mechanism may be related with mitotic spindles, myc targets, MTORC1 signaling, G2M checkpoint, E2F targets, DNA repair, glycolysis and unfolded protein response as GSEA identified. In addition, we further explored the prognostic value of *FAM64A* expression in different subgroups of pancreatic cancer and found that high *FAM64A* expression correlated significantly with G1/G2, stage I/II, and N1 cases.

At present, surgery is the only curable treatment for pancreatic cancer [20]. However, the possibility of recurrence, which adversely impacts patients’ outcomes, is an important factor in the choice of treatment. Here we also explored the correlation of *FAM64A* expression with recurrence, and found that this potential biomarker may help to guide treatment selection in pancreatic cancer patients. High expression of *FAM64A* also negatively affected OS and RFS among patients in with histological grade G1/G2 and clinical stage Ⅰ/Ⅱ cancers, but not with histological grade G3/G4 and clinical stage Ⅲ/Ⅳ cancers, which further demonstrates the specific prognostic role of *FAM64A* expression in subgroup analysis and its potential contribution to precision therapy for pancreatic cancer. However, the reduce number of patients evaluated in the later stages is a limitation in this study, future work need to expend the sample size to verify this conclusion.

Overall, our study provides evidence of the diagnostic and prognostic value of *FAM64A* expression in pancreatic cancer patients. However, the diagnostic and prognostic roles of high *FAM64A* expression were limited to stage Ⅰ and Ⅱ cases, and further identification of effective biomarkers in pancreatic cancer cases of advanced stage is imperative in the future.

## Conclusion

Our data demonstrate that *FAM64A* is upregulated in pancreatic cancer, and elevated *FAM64A* expression correlates with clinical progression and serves as an independent risk factor for OS and RFS in pancreatic cancer patients. Our findings suggest that *FAM64A* may be a useful biomarker in the diagnosis and prognosis of pancreatic cancer.
